# Recent Updates on Mechanisms of Resistance to 5-Fluorouracil and Reversal Strategies in Colon Cancer Treatment

**DOI:** 10.3390/biology10090854

**Published:** 2021-08-31

**Authors:** Shamin Azwar, Heng Fong Seow, Maha Abdullah, Mohd Faisal Jabar, Norhafizah Mohtarrudin

**Affiliations:** 1 Department of Pathology, Faculty of Medicine and Health Sciences, Universiti Putra Malaysia, Serdang 43400, Malaysia; shamin.azwar@gmail.com (S.A.); shf@upm.edu.my (H.F.S.); maha@upm.edu.my (M.A.); 2 Department of Surgery, Faculty of Medicine and Health Sciences, Universiti Putra Malaysia, Serdang 43400, Malaysia; faisal@upm.edu.my

**Keywords:** 5-fluorouracil, 5-FU, chemotherapy drug resistance, colon cancer, thymidylate synthase, thymidine phosphorylase, dihydropyrimidine dehydrogenase, methylenetetrahydrofolate reductase, overcoming chemotherapy drug resistance

## Abstract

**Simple Summary:**

Acquired resistance to chemotherapy by cancer cells is the predominant factor in chemotherapy failure, which ultimately leads to disease progression and death. Recent studies have presented compelling evidence of the various mechanisms and pathways through which cancer cells have developed resistance to drugs. This review summarises the mechanisms pertaining to 5-FU resistance and discusses ongoing efforts to prevent chemotherapy resistance in cancer cells and to re-sensitise them to cancer drugs.

**Abstract:**

5-Fluorouracil (5-FU) plus leucovorin (LV) remain as the mainstay standard adjuvant chemotherapy treatment for early stage colon cancer, and the preferred first-line option for metastatic colon cancer patients in combination with oxaliplatin in FOLFOX, or irinotecan in FOLFIRI regimens. Despite treatment success to a certain extent, the incidence of chemotherapy failure attributed to chemotherapy resistance is still reported in many patients. This resistance, which can be defined by tumor tolerance against chemotherapy, either intrinsic or acquired, is primarily driven by the dysregulation of various components in distinct pathways. In recent years, it has been established that the incidence of 5-FU resistance, akin to multidrug resistance, can be attributed to the alterations in drug transport, evasion of apoptosis, changes in the cell cycle and DNA-damage repair machinery, regulation of autophagy, epithelial-to-mesenchymal transition, cancer stem cell involvement, tumor microenvironment interactions, miRNA dysregulations, epigenetic alterations, as well as redox imbalances. Certain resistance mechanisms that are 5-FU-specific have also been ascertained to include the upregulation of thymidylate synthase, dihydropyrimidine dehydrogenase, methylenetetrahydrofolate reductase, and the downregulation of thymidine phosphorylase. Indeed, the successful modulation of these mechanisms have been the game plan of numerous studies that had employed small molecule inhibitors, plant-based small molecules, and non-coding RNA regulators to effectively reverse 5-FU resistance in colon cancer cells. It is hoped that these studies would provide fundamental knowledge to further our understanding prior developing novel drugs in the near future that would synergistically work with 5-FU to potentiate its antitumor effects and improve the patient’s overall survival.

## 1. Introduction

Colorectal cancer remains as the third leading cause of cancer-related mortality worldwide, with 1.8 million new patients diagnosed and 900,000 deaths reported annually [1]. Men are reported to be more susceptible to the disease, with 2.56 million cases reported in 2018 alone in comparison with the 2.19 million cases reported in women [2]. Most of these cases concern the elderly population, with 70% of cases occurring in individuals above 65 years of age [3]. Risk factors can either be attributed to environmental factors that include poor dietary habits, lack of physical activity, cigarette smoking, and alcohol consumption, or hereditary with pre-existing genetic predispositions.

Since its discovery in 1957, the infusional administration of 5-fluorouracil potentiated with leucovorin has been the mainstay preferred combinational chemotherapy option for solid tumors such as colorectal cancer as well as breast, stomach, and head and neck cancers [4,5]. For patients with advanced disease, 5-FU/LV combined with oxaliplatin (FOLFOX) or irinotecan (FOLFIRI) served as the first-line treatment with considerable improvement in patient’s relapse rate (RR) and overall survival (OS) despite the added toxicity. Being a fluorinated analog of uracil, 5-FU exerts its cytotoxic effects through the inhibition of thymidylate synthase (TYMS) that subsequently impairs cellular DNA synthesis functions in tumor cells [5]. Fluorinated byproducts of 5-FU metabolisms, that include fluorouridine triphosphate (FUTP) and fluorodeoxyuridine triphosphate (FdUTP) may also be misincorporated into RNA and DNA of tumor cells to further disrupt protein translation and expression. To further minimize the risk of disease relapse, targeted therapy with the use of agents such as anti-EGFR cetuximab and anti-angiogenesis bevacizumab may be additionally prescribed for patients in advanced stages [6]. Despite best efforts, the reported response rate of 5-FU as a single agent treatment is still fairly limited at between 10–15%. In comparison, the use of a FOLFOX or FOLFIRI regimen may see a significant improvement with reported objective response rate (ORR) of approximately 50% [7,8]. For patients treated with 5-FU-based regimens, the 3- and 5-year survival rates are reported to be at 72.2% and 60%, respectively [9,10]. However, considering that the first diagnoses of most patients are commonly of late presentations, it is alarming to note that the overall 5-year survival rate of these patients with advanced disease is still at a mere 18.3% [11]. It is thus crucial for more studies to be conducted to improve our best knowledge of the possible reasons behind 5-FU chemotherapy failure.

Chemotherapy failure that leads to disease progression and death is often the manifestation of chemotherapy resistance. Chemoresistance is defined by increased tumor cells tolerance against chemotherapy agents, as the pro-survival strategies against its directed cytotoxic effects. In this review, we will discuss new knowledge concerning the various mechanisms that may contribute to 5-FU resistance, particularly in colonic adenocarcinomas. These resistance mechanisms can be “classical”, as it may also concern other antineoplastic agents in contributing to the incidence of multidrug resistance (MDR), or “5-FU-specific”. We will also discuss recent approaches that are being investigated in the effort to enhance 5-FU sensitivity and overcome resistance.

## 2. Mechanisms of Action of 5-Fluorouracil

It was initially discovered that uracil is metabolized more rapidly than any other pyrimidine nucleotides in pre-neoplastic and hepatoma rats when compared to healthy rats [12]. The finding later encouraged a joint collaborative effort to synthesize a new class of antitumor compounds that could potentially exert its antitumor effects by targeting uracil metabolism in tumor cells [4]. The resulting compound is 5-FU as a heterocyclic compound with the structure almost identical to pyrimidine, but with a fluorine atom substituted from hydrogen at the C-5 position [13].

**Figure 1:**
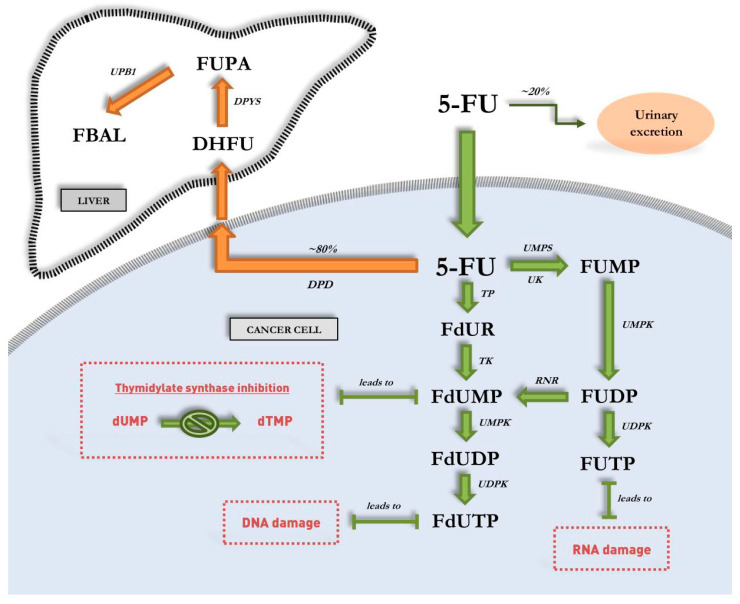
Various key enzymes facilitate the mechanism of action (anabolism) of 5-FU in cancer cells and its elimination (catabolism) via the hepatic system.

Following intravenous injection, only 1–5% of prodrug 5-FU is metabolized into active cytotoxic metabolites with approximately 20% being subjected to urinal excretion while the remaining 80% is rapidly degraded in the liver [14]. Degradation of 5-FU is facilitated by the enzyme dihydropyrimidine dehydrogenase (DPD) that catabolizes the conversion of 5-FU into 5,6-dihydro-5-fluorouracil (DHFU) as the inactive metabolite [15]. The catabolism process then continues with the conversion of DHFU to α-fluoro-β-ureidopropionic acid (FUPA) by dihydropyrimidinase (DPYS), and subsequently, its decarboxylation and deamination reactions to α-fluoro-β-alanine (FBAL) by β-ureidopropionase (UPB1). Anabolic pathway begins with the rapid entry of 5-FU into cells via a similar transport mechanism as uracil prior its conversion to either substrate fluorouridine monophosphate (FUMP) or fluorodeoxyuridine (FdUR) (Figure 1). Conversion to FUMP is catalyzed by the enzyme orotate phosphoribosyltransferase (OPRT) in the presence of phosphoribosyl pyrophosphate (PRPP). FUMP is then phosphorylated to fluorouridine diphosphate (FUDP), prior to another phosphorylation process that converts it to either active metabolite FUTP or into fluorodeoxyuridine diphosphate (FdUDP) by the enzyme ribonucleotide reductase (RNR) [16]. Being a fluorinated analogue of RNA nucleotide, FUTP can be misincorporated into the RNA of tumor cells and cause RNA damage. Given the central role of RNAs in protein translation, the protein expression of 5-FU-treated cells tend to be severely disrupted that promote the activation of cellular autophagy and apoptosis machinery [17]. In the meantime, FdUDP is further phosphorylated to active metabolite FdUTP and is misincorporated into the DNA of tumor cells to alternatively cause DNA damage. Prodrug 5-FU conversion to FdUR is facilitated by the enzyme thymidine phosphorylase (TYMP) prior to further phosphorylation to active metabolite fluorodeoxyuridine monophosphate (FdUMP). FdUMP may then form a stable ternary complex with enzyme TYMS along with 5,10-methylenetetrahydrofolate (CH_2_THF) as a methyl donor to irreversibly inhibit the enzymatic activity of TYMS [18]. This in turn prevents the conversion of substrate deoxyuridine monophosphate (dUMP) to deoxythymidine monophosphate (dTMP) that results in deoxynucleotide pool imbalances, and ultimately, in the arrest of cellular *de novo* DNA synthesis and repair.

## 3. Classical Mechanisms of Resistance

### 3.1. Alterations in Drug Transports

**Figure 2:**
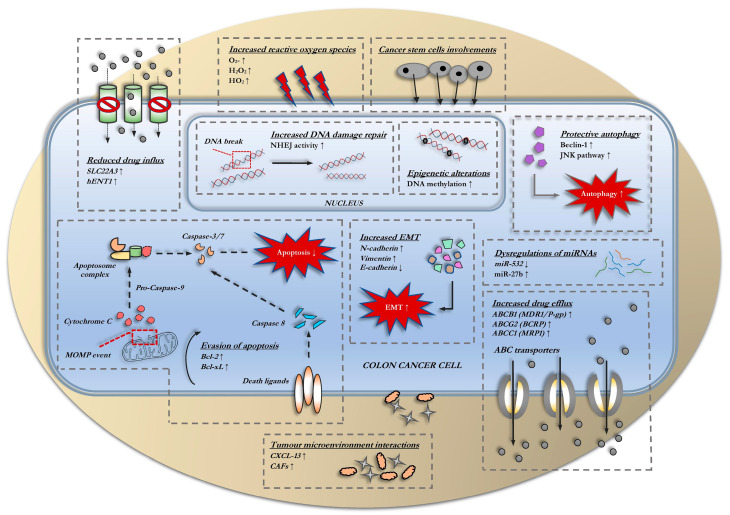
Recent discoveries on the classical drug resistance mechanisms involving 5-FU resistance in colon cancer cells.

Figure 2 summarizes the mechanisms of 5-FU resistance in colon cancer. The increased rate of intracellular drug exports reduces drug bioavailability within cells and is considered a major factor in the development of multi-drug resistance. Drug exports are predominantly facilitated by ATP-hydrolyzing, unidirectional transmembrane efflux pumps, notably, the ATP–binding cassette (ABC) transporters [19]. Overexpression of ABC proteins, however, presents an unfavorable implication on the prognosis of colon cancer patients receiving 5-FU treatment with studies reporting both negative and positive correlations [20,21,22,23]. Nonetheless, incidence of 5-FU resistance is still consistently motivated by elevated levels of ABC transporters, that includes ABCB1 [P-glycoprotein (P-gp) or MDR1] [24,25]; ABCG2 [breast cancer resistance protein (BCRP)] [26,27]; ABCC1 [multidrug resistance-associated protein (MRP1)] [28,29]; ABCC3 [20], and ABCC2 [30]. Surprisingly, in one study, 5-FU resistance in colon cancer cells was instead attributed to the loss of ABCB4 [31]. ABC transporters are substrate-specific that only conforms to structural changes upon binding with recognizable ligands facilitated by the hydrophobic transmembrane domain (TMD) [32]. Meanwhile, the nucleotide-binding domain (NBD), harnesses energy from ATP hydrolysis to allow these substrates to translocate across the cellular membrane. Studies that aim to explore the upstream influencers of ABC transporters remain scarce. It was determined that the transcription factor hairy and enhancer of split-1 (HES1) regulates the expression of ABCC1 and ABCC2 [30]. In other studies, ABCB1, ABCC1, and ABCG2 overexpression were found induced by not only transcription factor X-box binding protein (XBP1) but together with inositol-requiring enzyme 1 α (IRE1α), an endoplasmic-reticulum-localized protein that is activated upon endoplasmic reticulum stress [25]. CDK2-associated cullin domain 1 (CAC1) and antisense non-coding RNA (ncRNA) in the INK4 locus (ANRIL) influence on ABCB1 and ABCC1 were also demonstrated in recent studies, with ANRIL promoting their bindings with Let-7a microRNA (miRNA) precursor [27,33].

In addition to drug export, reduced drug accumulation within resistant cells may also be the outcome of decreased drug uptake. Unlike ATP-mediated drug efflux, the mechanism of drug influx into tumor cells can simply be via passive diffusion and is facilitated by a group of proteins belonging to the solute carrier (SLC) transporter superfamily [34]. The entry of 5-FU as an analogue of nucleoside is primarily facilitated by the SLC28 family, or also known as the “concentrative nucleoside transporters” (CNTs), and the SLC29 family, or also known as the “equilibrative nucleoside transporters” (ENTs) [35,36]. To the best of our knowledge, only one study had been conducted to investigate the link between these nucleoside transporters with 5-FU chemoresistance in colon cancer. It was concluded that high human ENT 1 (hENT1) level in tumor tissue is correlated with poor clinical response to 5-FU, supported by in vitro findings [37]. Similar findings were later observed in studies concerning pancreatic cancer, whereby, overexpression of hENT1 were associated with lower 5-FU chemosensitivity [38]. Surprisingly, the expression of SLCs such as SLC37A1, SLC22A3, and SLC39A7 were raised in tumors of colon cancer patients and are associated with poor patient prognosis and disease progression [39,40,41]. Higher SLC expression may have allowed for enhanced nutrient intake to support accelerated tumor growth.

### 3.2. Evasion of Apoptosis

Apoptosis or programmed cell death is a regulated form of cell death triggered upon exposure to irreversible damage or upon cellular senescence. In tumorigenesis, apoptosis is suppressed in an orchestrated manner through upregulations of anti-apoptotic proteins B-cell lymphoma 2 (Bcl-2), B-cell lymphoma-extra large (Bcl-xL), B-cell lymphoma-w (Bcl-W), and induced myeloid leukaemia cell differentiation protein (Mcl-1), and the downregulation of pro-apoptotic proteins Bcl-2 associated X protein (Bax), Bcl-2 interacting protein (Bim), Bcl-2 homologous antagonist/killer (Bak), BH3-interacting domain death agonist (Bid), and NOXA in the intrinsic mitochondrial apoptotic pathway to promote sustained tumor growth [42,43,44,45,46]. The equilibrium between these pro-apoptotic and anti-apoptotic proteins modulate the gateway of apoptosis and is implicated in drug resistance. Resistance to 5-FU typically involves the activation of nuclear factor kappa-light-chain-enhancer of activated B cells with signal transducer and activator of transcription 3 (NF-κB/STAT3) signaling pathway that not only promote the expression of anti-apoptotic Bcl-2, X-linked inhibitor of apoptosis (XIAP), and inhibitor of apoptosis protein (IAP) survivin, but also the expression of anti-proliferative proteins cyclin D1, vascular endothelial growth factor (VEGF), and c-Myc [47,48,49]. These 5-FU-resistant cells exhibited higher levels of Bcl-2, Bcl-xL, Mcl-1, and XIAP expression by which re-sensitization to 5-FU has been achieved through its inhibition which subsequently promotes the upregulation of Bax and Bcl2-associated agonist of cell death (Bad) expression [11,50]. Mechanistically, the activation of Bcl-2 and Bcl-xL expression drive constant retrotranslocation of pro-apoptotic proteins Bax and Bak away from the mitochondria into the cytosol to prevent its oligomerization that may result in mitochondrial outer membrane permeabilization (MOMP) [51]. MOMP event will release cytochrome C from the mitochondria into the cytosol and trigger a cascade of caspase-mediated apoptosis involving pro-caspase-9, and executioner caspase-3/7. Accordingly, pro-apoptotic BAX expression was suppressed in resistant cells [52]. Recent studies have also associated phosphoinositide 3-kinases with protein kinase B (PI3K/AKT) signaling pathway activation with upregulation of Bcl-2 and caspase-3, together with the downregulation of Bax and cleaved-caspase-3 with decreased tumor cell sensitivity towards 5-FU cytotoxicity [53]. Interestingly, these pathways may have been triggered concurrently as demonstrated in 5-FU-resistant LS174 colon cancer cells with the activations of JAK/STAT3, MAPK, PI3K/AKT, and NK-κB signaling pathways altogether [28]. Bcl-2 related ovarian killer (BOK) is another pro-apoptotic protein that has been recently-characterized. In their studies, Srivastava and colleagues (2019) had reported BOK to be a positive regulator of uridine monophosphate synthase (UMPS) in the metabolism of 5-FU, and that BOK inhibition had resulted in decreased tumor cell sensitivity towards 5-FU treatment [54]. Accordingly, patients receiving 5-FU therapy observed diminishing levels of BOK protein, suggesting a feedback mechanism triggered by tumor cells for survival.

Resistance may also be mediated through the extrinsic apoptosis pathway that triggers cascading caspase activation via cell-surface death receptors. The extrinsic pathway is pro-caspase-8-dependent, and its regulations in colon cancer involve the p53-mediated activation of death factors such as Fas and tumor necrosis factor alpha (TNF-α) [55]. Tumor cell tolerance against chemotherapeutic agents is usually the consequence of intrinsic pathway activation rather than extrinsic, albeit certain studies have demonstrated otherwise [56,57]. In a study, decreased expression of Fas, Fas ligand (FasL), and pro-caspase-8 were determined in colon cancer cells upon 5-FU treatment, suggesting the promotion of resistance through the extrinsic pathway [58]. Indeed, reduced levels of Fas and pro-caspase-8 were observed in 5-FU-resistant colon cancer cells [59,60]. Interactions by binding between Fas and FasL allow the recruit of adaptor protein Fas-associated death domain (FADD) that triggers the activation of pro-caspase-8, and subsequently, executioner caspase-3/7 that initiates the apoptosis process. Interestingly, Fas/FasL dysregulation also allows tumor cells to remain unrecognized by Fas^+^ lymphocytes and evade immune response [61]. Diminishing Fas expression coupled with CD133^+^CD24^lo^ tumor cells phenotype is correlated with decreased survival of colon cancer patients [62].

### 3.3. Changes in Cell Cycle and DNA-Damage Repair Kinetics

In eukaryotic cells, cellular proliferation and division can be divided into five well-characterized phases; G_0_ (cellular quiescence), G_1_ (physical and organelles developments), S (DNA synthesis), G_2_ (protein expression production), and M (mitotic division) [63]. The transition between phases is coordinated by oscillating levels of cyclins and cyclin-dependent kinases (CDKs) that act as “molecular checkpoints” responsible for the maintenance of genomic integrity and stability throughout the cell cycle. It is established that 5-FU exerts its cytotoxic effects in tumor cells primarily through inhibition of DNA synthesis. This is evident in the significant arrests of G_1_/S and S phases of cell cycle, as well as reduced G_2_/M phase population in colon cancer cells post-5-FU treatment [28,64]. 5-FU-resistant cells typically exhibit attenuated effects of 5-FU cytotoxicity with a higher population of cells detected in both G_1_/S and S phases when compared to 5-FU-susceptible cells [27,28,65,66]. When DNA synthesis remains unimpaired, 5-FU-resistant cells may also observe a higher population of G_2_ phase cells when compared to 5-FU-susceptible cells [26]. Prolonged G_1_ and S phases may provide sufficient time for tumor cells to counteract against 5-FU-induced DNA damage through activation of DNA repair pathways.

Progression of cell cycle is generally dependent on the absence or presence of DNA-damage response (DDR). It is especially pertinent in 5-FU treatment whereby FdUTP misincorporation into DNA bases may trigger base excision repair (BER) and the mismatch repair (MMR) pathways activation to excise false nucleotides from sequences. This in turn collapses the DNA replication forks to induce lesions that are described as double-stranded DNA break (DSB) resulting in DDR [67]. Two major pathways for DSB repair have been characterized, namely, the homologous recombination (HR) and the non-homologous end-joining (NHEJ) pathways. NHEJ typically serves as the major DSB repair pathway that is presented in all cycle stages, while HR functions are only limited to the late S and G_2_ phases of the cell cycle [68]. Inhibition of HR via targeting Rad51, an essential protein for DSB repair, led to an enhancement of 5-FU response [69].

In 5-FU-resistant cells, NHEJ activities are reported to be higher via the upregulation of its mediators that ultimately contribute to increased DNA damage repair and resistance to apoptosis [70,71]. These responses, nonetheless, are orchestrated by members of the PI3K family, namely, ataxia-telangiectasia mutated (ATM) and ATM- and Rad-related (ATR) kinases [72]. These kinases facilitate the recruitment of repair mediators into the DNA-damaged sites while stalling cell cycle progression in G_1_, S, or G_2_ phases through the activation effector checkpoint kinase (Chk1 and Chk2). It has been demonstrated that DNA damage invoked by 5-FU treatment activates ATM/ATR-mediated Chk1/Chk2 upregulation that leads towards S and G_2_M phases cell cycle arrest, respectively, and ultimately in cellular apoptosis [73,74,75,76]. Conversely, the absence of ATM/ATR-mediated Chk1/Chk2 activations is observed in 5-FU-resistant that hinders apoptosis.

### 3.4. Involvement of Autophagy

When under metabolic or replication stress, tumor cells may also undergo autophagy that sequesters damaged organelles or proteins into lysosomes for degradation as the protective catabolic mechanism to prevent further cellular damage and to maintain cellular homeostasis [77]. Replication stress that is induced by 5-FU therapy typically reflects in pro-survival autophagy response in 5-FU-resistant cells marked by increased activations of Beclin-1 and microtubule-associated proteins 1A/1B light chain 3B II (LC3-II) expression, following mammalian target of rapamycin (mTOR) pathway activation [78,79,80]. Beclin-1 facilitates the formation of LC3-II autophagosome from LC3-I that interacts with phosphatidylethanolamine (PE), autophagy-related genes 3 (Atg3), and Atg7 to allow for binding and degradation of erroneous substrates induced by 5-FU treatment [77]. Interestingly, there are growing number of studies that had attribute 5-FU resistance with autophagy inhibition instead [81,82,83,84]. It is due to these contraindicatory data that the therapeutic potential of autophagy modulation is still a subject of ongoing debates as autophagy involvement can be both pro-survival and pro-death. It is postulated that autophagy inhibition serves as the protective mechanism for resistant cells to avoid autophagic cell death [85].

### 3.5. Epithelial-to-Mesenchymal Transition (EMT)

A hallmark of cancer progression and metastasis is the increased capability of cancer cells to migrate and invade neighboring and distant tissues. This is commonly achieved following a morphogenetic process termed as epithelial-to-mesenchymal transition (EMT) in which epithelial cells losses its epithelial traits and acquire mesenchymal properties through cytoskeleton remodeling [86]. EMT is not only a well-known risk factor that is associated with tumor metastasis in the liver of colon cancer patients that leads to poor patient prognosis but is also associated with poor treatment response [87,88,89,90]. Over the years, growing literature has demonstrated the link between EMT events and 5-FU resistance as higher and reduced expression of mesenchymal and epithelial markers, respectively, were consistently reported in 5-FU-resistant cells. In these studies, levels of vimentin, zinc finger protein SNAI1 (SNAIL), phosphorylated nuclear factor NF-κB p65 subunit (p–p65), ten-eleven translocation methylcytosine dioxygenase 1 (TET1), and naked cuticle 2 (NKD2) were found to be significantly upregulated [48,91,92]. In contrast, the expression of E-cadherin, β-catenin, transcription factor 4 (TCF4), and Axin were downregulated. The transition can be mediated through several signaling pathways that include NF-κB, Wnt, and Akt following stimulation from transcription factors [e.g., twist-related protein 1 (TWIST1) and zinc finger E-box-binding homeobox 2 (ZEB2)], non-coding RNAs [e.g., metastasis-associated lung adenocarcinoma transcript 1 (MALAT1), SLC25A25-AS1, and miR-23b), and epigenetic alterations (e.g., promoter methylation of TWIST1/2, ZEB2, and SNAI1/2) [93,94,95,96,97,98]. Interestingly, the activations of these EMT factors may also influence the regulation of other resistance mechanisms. For instance, the overexpression of SNAIL has been demonstrated to be capable of upregulating the expression of ABC transporter, ABCB1, to promote resistance [24]. Other studies have also reported increased stemness of tumor cells following the activation of EMT factors TWIST1 and ZEB2 [98,99].

### 3.6. Involvement of Cancer Stem Cells

Cancer stem cells (CSCs), also known as “tumor-initiating cells”, are a small subpopulation of cancer cells within tumors that have attained stem cell-like characteristics such as self-renewal and multi-directional differentiation capabilities [100]. There is growing evidence demonstrating the pivotal role of CSCs in tumor initiation, progression, metastases, and cancer recurrence, aside from contributing to intra-tumor heterogeneity [101,102,103,104]. CSCs are poorly differentiated in nature and possess the ability to remain quiescent in the G_0_ phase of the cell cycle, thus, helping them to escape from chemotherapy insults, which typically targets highly-proliferating and mature cancer cells [105]. Recent studies have also demonstrated the ready capability of CSCs to promote EMT via transcription factor ZEB2 even when untreated [99]. Furthermore, colon CSCs when under chemotherapy stress, may directly prompt the activation of other resistance pathways. These include raised Bcl-2 and Bcl-xL that inhibit apoptosis [106,107]; higher expression of ABC transporters such as ABCC2, ABCC3, and ABCG2 that promote drug export from the cell [20,108]; overexpression of DNA repair gene O^6^-methylguanine-DNA-methyltransferase (MGMT) [109,110]; and the alteration of cell cycle checkpoint via phosphorylation of ATM, Chk1, and Chk2 proteins [111]. Akin to other 5-FU-resistant colon cancer cells, colon CSCs may also promote 5-FU resistance within the tumor microenvironment via the activation of PI3K/AKT signaling pathway that regulates cell growth and apoptosis [112,113]. Wnt signaling also plays a critical role in mediating drug resistance in CSCs as p300/β-catenin binding has been shown to promote differentiation while CREB-binding protein/β-catenin binding is necessary for potency maintenance of CSCs [114,115]. The knockdown of β-catenin may not only result in increased drug sensitivity through diminishing levels of CSCs’ stemness but also in the inhibition of EMT activities [116].

Indication on the plausible involvement of colon CSCs to 5-FU resistance was first implied when isolated colon cancer cells presented with colon CSCs marker CD133 showed increased resistance towards apoptosis following 5-FU exposure when compared to CD133^–^ cells [117]. Indeed, as demonstrated in recent studies, these CD133^+^ together with CD44^+^ cells do exhibit CSC-like phenotypes with increased viability, colony formation, migration, and invasion rates, alongside resistance towards apoptosis [104,118,119]. Interestingly, it has been shown that only a small subset of CD133^+^ cells is implicated with 5-FU resistance, such as in CD133^+^CD24^lo^ cells [120]. Nonetheless, higher expression of CD133 has been correlated with poor prognosis in stage II and III colon cancer patients [121,122]. In contrast, a higher level of CD44 presents an increase in risk for colon cancer as well as a worse overall survival of patients [123,124,125]. Aside from CD133, CD44, and CD24, the knockdown of other putative colon CSCs stemness such as aldehyde dehydrogenase (ALDH1) [126] and CD166 [127,128] have also exhibited increased cellular 5-FU-mediated cytotoxicity.

CSCs are also a major determining factor that contribute towards intra-tumoral heterogeneity. In theory, it is postulated that CSCs are placed at the apex of the division hierarchy, capable of undergoing both symmetric and asymmetric divisions that may differentiate into different types of cancer cells when triggered by environmental stimuli [129]. This results in a phenomenon known as clonal evolution, by which a single tumor may feature distinctive subpopulation of tumor cells carrying a wide range of genetic variation. 5-FU therapy is an example of an environmental stimulus that may drive tumor evolution. In one study, it was shown that 5-FU treatment promotes T>G mutation in human small intestinal organoids cultures, causing subsequent 5-FU treatment to become less cytotoxic [7]. The mutation is perhaps a pro-survival feedback mechanism for the tumor cells to adapt to the cytotoxic environment. To make things more complicated, clonal variation also accounts for tumor protein 53 (p53) mutational status in patients that have likewise been shown to influence 5-FU chemosensitivity [130].

### 3.7. Interactions within the Tumor Microenvironment

Almost all mammalian cells, including tumor cells, are in constant communication with their respective microenvironment for the maintenance of cellular homeostasis and general survival [131]. For tumor cells, its interaction with its tumor microenvironment (TME) such as with supporting extracellular matrix (ECM) [132,133]; neighboring stromal cells which include cancer-associated fibroblasts (CAFs) [134], mesenchymal stem cells (MSCs) [135], and blood and lymphatic networks [136,137]; together with immune cells such as tumor-associated macrophages (TAMs) [138,139], natural killer cells (NK cells) [140,141], and T/B lymphocytes [142,143,144] have been the highlights of many researchers due to its fundamental role in colon cancer progression and metastasis. The significance of TME in mediating 5-FU drug resistance has also gained attention in recent years due to its crosstalk influence on tumor cell behavior. For instance, in one study, chemokine C-X-C motif ligand 13 (CXCL-13) is found highly-expressed in the TME of 5-FU-resistant colon cancer cells as well as in sera of 5-FU-resistant patients associated with worse clinical outcome [145]. Although the mechanism as to how CXCL-13 participates in 5-FU resistance was not stated, it is highly plausible that resistance is achieved through piggybacking the same pathways responsible in promoting tumor growth, migration, and invasion in CXCR5-expressing colon cancer cells; through the activation of PI3K/AKT/mTOR and Wnt/β-catenin signaling pathways [112,146,147]. Nonetheless, it was shown that CXCR-5 knockout mice exhibited an increased number of infiltrating B-cells that had contributed towards improved drug response. Meanwhile, a direct connection between CXCL12/CXCR4 axis and Wnt/β-catenin signaling in enhancing 5-FU resistance can be observed in other studies [148,149,150]. CXCL-13 and CXCL-12, as chemokines, may also recruit T-regulatory cells into the microenvironment that would aid in 5-FU tolerance. This is evident in the CCL20/CCR6 axis as its overexpression in resistant patients has been associated with increased TFCD4^+^ infiltration from tumor-infiltrating lymphocytes (TILs) via forkhead box protein O1 (FOXO1)/CEBPB/NF-κB pathway [151]. Secreted transforming growth factor beta (TGF-β) may further facilitate resistance by prompting SMAD3 nuclear translocation that promotes neovascularization [152]. A higher level of TGF-β may also promote TAMs polarization, specifically by M2 macrophages that secrete CCL22 to confer 5-FU resistance via the PI3K/AKT pathway [153,154]. Paradoxically, improved disease-free survival is observed in stage III colon cancer patients expressing a high level of M1 macrophages, suggesting a synergistic effect through immunogenic death with 5-FU via TNFα/TRAIL [155].

Tumor cells also interact with other stroma cells, such as with CAFs that actively partake in ECM deposition and remodeling despite their dual-role as both pro-tumorigenic and tumor-suppressive [156]. Aside from recruiting TAMs, transformed CAFs may secrete cytokines such as IL-6, IL-8, IL-17, TNF-α, and VEGF that does not only contribute towards tumor aggressiveness but also in therapy response such as in 5-FU treatment [157,158,159]. Based on in vitro data, survival advantage gained can be attributed to AKT, mitogen-activated protein kinases 14 (P38), and Survivin nuclear translocation; elevated expression of CD44, β-catenin, leucine-rich repeat-containing G protein-coupled receptor 5 (LGR5), and ABCG2; STAT3 augmentation; alongside PI3K/AKT and Janus kinase (JAK)/STAT pathways activation. CAFs may also promote tumorigenesis and 5-FU resistance through generating CSCs, resulting in a plethora of resistance pathways previously discussed [158,160]. While CAFs appear as an excellent target for anticancer drugs, conflicting data have also been presented in recent years that strongly suggest its role as a tumor suppressor [161,162].

As a molecular vehicle that exports a selective repertoire of DNA, RNA, proteins, lipids, and metabolites from one cell to another, the role that exosomes as extracellular vesicles play in mediating communication between tumor and stroma cells is undeniably extensive. In recent years, circulating exosomes have been demonstrated to mediate 5-FU resistance through the transfer of CAFs secretomes [163], transcription factor phosphorylated STAT3 (p-STAT3) [164], glycoprotein Wnt [165], circular RNAs circ_0000338 [166], as well as microRNAs such as miR-210 [167], miR-21 [168], and miR-145 [169]. Furthermore, exosomal marker tumor-associated glycoprotein 72 (TAG72) have been correlated with 5-FU-resistant patients, indicating its prognostic potential as a novel, non-invasive evaluation tool to predict patient’s response to 5-FU-based therapy [170].

### 3.8. Epigenetic Alterations

Epigenetic modification refers to the heritable changes that do not directly affect the DNA sequence *per se*, but rather its accessibility and chromatin structure, attributed to incidences of DNA methylation and histone modifications that lead to the dysregulation of gene expression [171]. In colon cancer, dysregulation that elicits genomic instability causing tumor initiation can derive from gene promoter CpG island methylation as observed in tumor-suppressors Krüeppel-like factor 6 (KLF6), KLF4, and zinc finger protein 726 (ZNF726) silencing; through direct hypermethylation of one allele demonstrated in human mutL homolog 1 (hMLH1) and cyclin-dependent kinase inhibitor 2A (CDKN2A); and through DNA hypomethylation such as in the case of LINC00460 that promotes metastasis [172,173,174,175].

Certain methylation processes may also contribute to incidence of 5-FU resistance, aside from tumor progression. It was shown that protocadherin-17 (PCDH17) silencing via promoter methylation does not only suppresses its role as a tumor-suppressor in inducing apoptosis, but also in inducing JNK-dependent autophagic cell death upon 5-FU treatment in colon cancer [83]. Higher expression of PCDH17 is thus correlated with better OS of 5-FU-treated patients. In one study, integrated analysis of methylation profiling and protein-protein interaction (PPI) revealed that 5-FU resistance conferred through promoter hypermethylation can be a multitude, ranging from the involvement of p53 and epidermal growth factor receptor (EGFR) signaling pathways (e.g., EGFR and IGFBP3), drug metabolism through cytochrome P450 [e.g., cytochrome P450 3A5 (CYP3A5) and glutathione S-transferase P (GSTP1)], as well as in pyrimidine metabolism [e.g., cytidine deaminase (CDA)] [176]. Compelling evidence has been presented that linked miR-181a/135a/302c promoter hypermethylation, 5-FU tolerance, and microsatellite instability/microsatellite stable (MSI/MSS) status in colon cancer cells. In their studies, Shi and colleagues (2018) had demonstrated significant CpG island hypermethylation in promoter regions of miR-181a/135a/302c in cancer tissues of MSI patients when compared to MSS patients [177]. Interestingly, demethylation in vitro exerted reinforced 5-FU sensitivity in MSI phenotypic cells via targeting pleiomorphic adenoma gene 1 (PLAG1). Although most studies have reported negative correlations between gene hyper-/hypo-methylation and poor patient prognosis through resistance, such as in BCL2/adenovirus E1B 19 kDa protein-interacting protein 3 (BNIP3) facilitated by DNA methyltransferase 1 (DNMT1) enzyme [178]; miR-26b via P-gp down-regulation [179]; DNMT3A and DNMT3B overexpression [180]; CCNE1, cyclin D1 binding protein 1 (CCNDBP1), paraoxonase 3 (PON3), as well as DEAD-box helicase 43 (DDX43) and cell adhesion molecule L1-like I (CHL1) via the mitogen-activated protein kinase (MAPK) apoptosis and PI3K/AKT proliferation pathways, respectively [181]; and osteopontin splicing isoform c via methyl-CpG binding protein 2 (MeCP2) [182], there are studies that had reported otherwise; WNT5A [183]; and NME/NM23 nucleoside diphosphate kinase 2 (NME2) [184]. Histone modifications are another common cause for epigenetic alterations and can be observed in the histone methylation H3K9me9 dysregulation and H3K27 PCAF-mediated histone acetylation of p53 [66,185]. Histone deacetylases are essential enzymes required for the reversible acetylation of histone. Its degradation via deubiquitinase USP38, specifically by histone deacetylase 3 (HDAC3), is also implicated in 5-FU resistance as epigenetic regulation via H3K27 acetylation is implied [186].

### 3.9. Dysregulations of miRNAs

MiRNAs are small, non-coding RNA molecules that have been implicated in various biological processes including colon tumorigenesis due to its capability to regulate gene expression post-transcriptionally via base-pairing with complementary mRNA. These micRNAs include miR-20a, -182, -122, -425-5p, -221, -200, and -215-3p, that amongst others, target TGF-β, nucleic acid-binding protein 1 (NABP1), fructose-biphosphate aldolase (ALDOA), FOXO, quaking homolog, KH domain RNA binding (QKI), Ras association domain family member 2 (RASSF2), and forkhead box M1 (FOXM1) to influence tumor cell proliferation, migration and invasion, apoptosis, cell cycle, as well as DNA-damage repair [187,188,189,190,191,192,193]. A plethora of miRNA species that regulates tumor cell response towards 5-FU in colon cancer have also been described over the years (Table 1). MiR-532, for instance, may bind directly to the 3′UTR regions of ETS proto-oncogene 1 (ETS1) and transglutaminase 2 (TGM2) to suppress its expression and inhibit further Wnt/β-catenin signaling activation [194]. When factoring in p53 activation that is also induced by miR-532-3p, significant restoration in 5-FU sensitivity can be observed attributed to increased cell cycle arrest and early apoptosis. Bioinformatic analysis revealed miR-494 to be correlated with 5-FU resistance through DNA topoisomerase IIA (TOP2α) overexpression [195]. Although the mechanism of resistance was not stated, the role of TOP2α that maintains the topological status of chromosome during DNA replication and transcription may have facilitated enhanced tumor cell proliferation and DNA-damage repair in 5-FU-resistant cells [196].

Regulation of miRNAs can be influenced by other genes and proteins. For instance, reduced levels of miR-125b have been demonstrated to result in toll-like receptor 2 (TLR2)/6 and TLR5 overexpression [214]. Subsequently, miR-125b-5p downregulation induces specificity protein 1 (Sp1)-mediated activation of CD248 that does trigger not only tumor cells metastases but also 5-FU chemoresistance. Up-regulation of lncRNA POU6F2-AS2 is shown to inhibit miR-377 expression, leading towards BRD4 upregulation which in turn promotes tumor cell proliferation, cell cycle progression, and reduced 5-FU cytotoxicity [211]. In a different study, sponging miR-139-5p attributed by LINC00152 overexpression has been linked to diminishing 5-FU-induced apoptosis in colon cancer cells via notch homolog 1, translocation-associated (NOTCH1), Bcl-2, and autocrine motility factor receptor (AMFR) regulations [212]. Recent studies on other microRNAs include miR-27b-3p that acts as a tumor-suppressor. By regulating ATG10, a protein essential for autophagosome formation, it markedly increases the sensitivity of colon cancer cells to 5-FU in vivo [213].

### 3.10. Redox Imbalances

The role reactive oxygen species (ROS) plays in regulating various signaling pathways that may initiate tumorigenesis, differentiation, and apoptosis have been widely documented [215]. Elevated levels of ROS due to redox imbalance characterized by increased free radical hydroxyl radical (•OH), hydrogen peroxide (H_2_O_2_), hydroperoxyl radical (HO_2_•), and superoxide anion (O_2_•^−^) can induce DNA damage, cell cycle arrests, as well as autophagy via oxidative stress [216,217]. ROS may also influence tumor cell response to chemotherapy. In a recent study, the inhibition of nuclear factor erythroid 2-related factor 2 (Nrf2) as the key transcription factor in the regulation of cellular redox homeostasis has resulted in the re-sensitization of 5-FU in colon cancer cells [218]. Nrf2 inhibition triggered by FoxO3 upregulation caused a significant reduction in TR1 expression that in turn, elevates intracellular ROS. Synergistic interaction between ROS with 5-FU to further trigger apoptotic cell death in resistant cells was further supported in another study by Yan and colleagues (2019) [217]. It was determined that increased intracellular ROS had induced AMP-activated protein kinase (AMPK) signaling pathway activation that promotes autophagy initiation and reverse 5-FU resistance in colon cancer cells.

## 4. Mechanism of Resistance by Key 5-Fluorouracil Enzymes

### 4.1. Amplification of Thymidylate Synthase

Inhibition of thymidylate synthase enzymatic activity has been the hallmark cytotoxic mechanism of 5-FU treatment. Under normal physiological conditions, TYMS is responsible for the irreversible methylation of dUMP to dTMP with the aid from CH_2_THF as the methyl donor [219]. The absence of TYMS thus, hinders the production of nucleolar thymidine as an essential DNA nucleoside, leading towards *de novo* DNA synthesis impairment within cells. Given that tumor cells are associated with accelerated rates of DNA replication and repair due to increased genomic instability, it is thus more affected than healthy cells [220]. A growing amount of evidence has presented the prognostic value of TYMS, as reduced expression denotes improved patient response and OS to 5-FU-based therapy [221,222]. This corresponds to early studies by which an inverse relationship is observed between TYMS expression and 5-FU chemosensitivity [223,224]. Accordingly, increased TYMS amplification has been recognized as a primary determinant in the development of 5-FU resistance (Table 2) [225].

Underlying mechanisms leading towards TYMS amplification have been attributed to the incidence of copy number variation (CNV) and tandem repeat polymorphism within the 5′-untranslated regions (5′-UTR) of TYMS gene. Due to its polymorphic nature, the 5′-UTR region is inclined to contain a double (2R) or triple (3R) 28 base-pair tandem repeats, as well as G > C single nucleotide polymorphism (SNP) that may significantly affect TYMS translation. In recent studies, 5-FU chemotherapy efficacy was found markedly reduced by 50% in patient cohort afflicted with 5′-UTR 2R/3G, 3C/3G, and 3G/3G polymorphisms, when compared to 5′-UTR 2R/2R, 2R/3C, and 3C/3C carriers [226]. While in separate studies, the loss of heterozygosity (LOH) was factored in as well, reporting lower risk of disease recurrence and death for patients with 2G/3G, 2G/LOH, and 3C/LOH genotypes [227]. Although no correlation between 3′-UTR polymorphism and 5-FU efficacy was found in the study conducted by Qihong Nie and colleague (2019), it was established by Ntavatzikos and colleagues (2019) that the presence of 3′-UTR ins/LOH is an independent indication for increased risk for disease relapse and death. The phenomenon of TYMS copy number gain upon 5-FU administration in colon cancer patients has also been reported, suggesting the mechanistic approach behind acquired 5-FU resistance is through selective pressure [7]. It is plausible that TYMS overexpression is the negative feedback response of tumor cells to overcome the competitive inhibitory binding of FdUMP as the active substrate of 5-FU.

### 4.2. Suppressed Expression of Thymidine Phosphorylase

Thymidine phosphorylase, or also known as platelet-derived endothelial cell growth factor (PD-ECGF) is another crucial enzyme in the metabolism of 5-FU, involved in the conversion of 5-FU to FdUR prior to its phosphorylation by thymidine kinase (TK) to FdUMP [241]. TYMP overexpression may promote increased intracellular concentration of active 5-FU metabolite, thus enhancing its efficacy. Even so, its direct correlation in 5-FU treatment outcome was previously a subject of debate as certain studies linking elevated expression with worse patient prognosis [242,243,244]. Recent studies later conclusively determined that TYMP overexpression may only benefit early-stage patients in prolonging time to disease progression, relapse-free survival, and OS [229,230,231,232,233,245]. This is supported by experimental studies in which enhanced 5-FU cytotoxicity is observed in conditions where TYMP is upregulated in colon cancer cells and xenograft mice [234,246]. Accordingly, reduced levels of TYMP may contribute to the development of acquired 5-FU resistance [247]. Poor patient outcomes observed in advanced-stage patients despite elevated levels of TYMP may be attributed to the dual role of TYMP in promoting angiogenesis and metastasis, aside from its role in 5-FU metabolism [222,231]. Such discrepancy may be the result of contrasting treatment regimens between primary and metastatic tumors as the administration of oxaliplatin, irinotecan, and capecitabine alongside 5-FU may provoke TYMP expression [248,249].

Little to no evidence has been presented to attribute TYMP dysregulation with genetic changes of TYMP gene [250]. Genetic variations of TYMP are generally linked with the incidence of mitochondrial neurogastrointestinal encephalomyopathy syndrome (MNGIE) as an autosomal recessive disorder that affects the gastrointestinal and nervous systems [251]. The condition is due to the loss of function of TYMP gene following homozygous mutations c.1283 G > A, and c.1284 T > A, as well as mutations in DNA polymerase subunit gamma (POLG) and ribonucleotide reductase regulatory TP53 inducible subunit M2B (RRM2B) genes that lead to TYMP deficiency [252,253,254]. Interestingly, increased TYMP expression has been reported in cases of local inflammations such as rheumatoid arthritis and psoriasis, suggesting its plausible causal link with stress conditions such as hypoxia in the tumor microenvironment [255,256]. Indeed, TYMP overexpression may promote adaptive responses mediated by the hypoxia-inducible factor (HIF-1) pathway that prevents hypoxia-induced apoptosis that is triggered upon continuous chemotherapy [229,242,257]. Consequently, attenuation of 5-FU chemosensitivity can be observed across varying tumor cells exposed to hypoxic conditions, including colon cancer cells [258,259,260].

### 4.3. Overexpression of Dihydropyrimidine Dehydrogenase

Dihydropyrimidine dehydrogenase serves as the initial rate-limiting enzyme in the catabolism of prodrug 5-FU to inactive DHFU and is responsible for approximately 80% of 5-FU degradation in the liver after bolus infusion. Consequently, DPD moderates the bioavailability and concentration of plasma 5-FU that inversely correlates with 5-FU treatment response in treated patients. In DPD-deficient patients, increased risk and severity of 5-FU-induced toxicity as a result of active 5-FU metabolites accumulation has been described [261,262]. Accordingly, increased intra-tumoral DPD expression may lead to reduced concentration of 5-FU metabolites and subsequently, in the development of 5-FU resistance [237,263]. This has been shown evidently in colon cancer cells where higher levels of DPD activity are observed in the liver of nude mice xenografted with 5-FU-resistant HT-29 cells when compared to nude mice xenografted with 5-FU-sensitive HT-29 cells [235]. Successful in vitro and in vivo reversal of 5-FU resistance in colon cancer cells through DPD inhibition has also been reported in recent studies [236]. Patients displaying heighten adverse events of 5-FU-induced toxicity are often screened for DYPD*2A polymorphisms, particularly in splice site variants 1905 + 1G > A, c.1601G > A (p.Ser534Asn), c.2194 G > A (p.Val732I1e) [264]. Corrected 5-FU treatment dose is accordingly administered to patients assessed positive for DYPD*2A mutations if the occurring adverse events are deemed intolerable. Despite the toxicity, certain DYPD mutations offer improved tumor response to 5-FU treatment (Table 3) [226].

### 4.4. Overexpression of Methylenetetrahydrofolate Reductase

Inhibition of TYMS is achieved through stable ternary complex formation between TYMS, FdUMP, and CH_2_THF. The availability of CH_2_THF for binding, however, is tightly-regulated by the enzyme methylenetetrahydrofolate reductase (MTHFR) that catalyzes the irreversible conversion of active CH_2_THF to 5-methylenetetrahydrofolate (5-MTHF) as the essential folate for DNA methylation [267]. Given that TYMS inhibition serves as the principal mechanism behind 5-FU cytotoxicity, MTHFR has been regarded as a factor that may influence the efficacy of 5-FU therapy. It is postulated that MTHFR underexpression may contribute towards enhanced 5-FU efficacy due to a higher concentration of CH_2_THF substrate while its overexpression may manifest in 5-FU resistance. Two common polymorphism variants with the outcome of MTHFR enzymatic deficiency have been identified, namely, the rs1801133 (C677T) and rs1801131 (A1298C) [269]. Positive correlation between MTHFR polymorphisms and colon cancer patient’s response to 5-FU treatment is often reported involving variant A1298C with C677T seeing 5-FU benefits only exclusively in refractory cases [239,270,271]. Interestingly, in vitro studies have implicated both polymorphisms in the development of 5-FU resistance in colon cancer cells [240]. Nonetheless, several studies had reported no correlation between MTHFR polymorphism and tumor response rate in early-stage patients, supported by in vitro models [272,273,274,275]. These variabilities may be contingent with interpatient variability in folate status and treatment history, as better treatment response was reported for MTHFR-mutated patients receiving 5-FU monotherapy when compared to patients with a history of FOLFOX or FOLFIRI treatment [274].

## 5. Reversal Strategies

Over the years, various approaches have been undertaken in attempts to promote increased colon cancer tumor cells’ sensitivity towards 5-FU treatment that would ultimately improve patient’s prognosis. Based on the resistance mechanisms discussed, it can be summarized that reduced levels of TYMS, DPD, MTHFR, as well as increased levels of TYMP would greatly benefit in 5-FU re-sensitization as these enzymes are directly involved in its metabolism and degradation. In the meantime, increased drug influx, apoptosis events, DNA-damage repair, and cell cycle progression, together with reduced drug efflux and autophagy activity would not only aid in reversing 5-FU resistance, but also in the incidence of MDR. Although seemed fairly straightforward, strategies to overcome resistance through actualizing these reverse mechanisms have remained a significant challenge owing to the high complexity and heterogeneity of tumor cells that are not just between individuals, but also in between tumor cells within the same microenvironment attributed to clonal evolution. A plethora of small molecule inhibitors (SMIs), non-coding RNAs, and plant-derived small molecules have nonetheless been investigated in recent years for its potential to overcome 5-FU resistance given the circumstances. Considerable advancements in the field of genomics, proteomics, and metabolomic through high-throughput technologies in recent years have also allowed for resistance profiling in between patients that would aid in personalized medicine to minimize the risk of chemotherapy failure.

### 5.1. Small Molecule Inhibitors

Table 4 summarizes the small inhibitors that can enhance 5-FU chemosensitivity. Most of the compounds described having worked synergistically with 5-FU to potentiate its cytotoxicity effects and overcome its resistance falls under the class of small molecule inhibitors. HDAC inhibitors such as depsipeptide and valproic acid (VPA) for instance, are capable of potentiating the antitumor activity of 5-FU in colon cancer via the induction of caspase-3/7 activation, MHC class II gene expression, cell cycle arrest by cyclin-dependent kinase inhibitor 1A (CDKN1A) upregulation, and extensive TYMS downregulation [275,276]. The use of VPA has however, been controversial due to its dual role functions of downregulating and upregulating TYMP and TYMS expression, respectively. Synergistic interactions between VPA and 5-FU were only observed in p53^wt^ and p53^mut^ colon cancer cells, but not in p53^–^ cells. Considering that TYMP upregulation may only benefit patients in earlier stages of cancer due to its adverse role in promoting angiogenesis in advanced stages and that p53^–^ cells are often associated with advanced stages, the use of VPA would appear paradoxical. Further downregulation of TYMS can also be achieved through co-treatment with mitogen-activated protein kinase kinase (MEK) inhibitor cobimetib, FOXM1 inhibitor thiostrepton, and heat shock protein 90 (HSP90) inhibitors such as luminespib and ganetespib [277,278,279,280,281]. Interestingly, U0126 as inhibitors of MEK may also restore 5-FU chemosensitivity through the generation of more γH2AX foci and diminishing the expression of DNA excision repair 1 (ERCC1), aside from suppressing TYMS expression [282]. Based on the resistance mechanisms discussed, certain SMIs recently studied may also be considered as potential agents to potentiate 5-FU cytotoxicity effects. Patients evaluated with higher expression of ABC transporters such as ABCC1, ABCB1, and ABCG2 for instance, may benefit significantly from 5-FU therapy when co-administered with uracil analogue U-332 that may abrogate the expression of all three transporters [283]. Alternatively, patients assessed with DPD overexpression commonly associated with incidence DYPD*2A polymorphisms may consider the use of 5-FU together with JTE-013 to effectively inhibit S1PR2 as the upstream regulator of DPD expression [236]. Gimeracil is another reversible inhibitor of DPD, known to be combined alongside oteracil potassium and tegafur as prodrug 5-FU, in a preparation simply termed as S-1 [284]. Stage III colon cancer patients receiving S-1 plus oxaliplatin reported a slight improvement in 3-year DFS when compared to patients receiving only tegafur-uracil plus leucovorin. Other small molecule inhibitors studied to be effective in reversing 5-FU in colon cancer cells may include phospholipase A2 inhibitor quinacrine that enhances c-Jun N-terminal kinase (JNK1)-dependent Nrf2 degradation, and DNA methyltransferase inhibitor decitabine that demethylates TYMP promoter (Table 4) [285,286]. For patients with advanced disease, TYMP inhibitor such as tipiracil may not only potentiate the antiproliferative effects of 5-FU but also impede the progression of metastasis [287]. Interestingly, tipiracil has already been actively-used alongside other molecules in the U.S. Food and Drug Administration (FDA)-approved compound TAS-102 for the treatment of metastatic colon cancer.

### 5.2. Plant-Derived Small Molecules

The high toxicity and lack of specificity of many synthetic resistance-reversing agents have also motivated studies to highlight the plausible potentials of the natural dietary bioactive compound such as flavonoids to combat drug resistance. In fact, nearly half of newly identified compounds and newly discovered drugs were derived from studies on natural products [298]. *Sophorae flavescens* or shrubby sophora, is a plant with a long history of use in traditional Chinese medicines to elicit antioxidants effects and immunity enhancement. When co-treated with 5-FU in colon cancer cells, compound Sophora injection had also improved 5-FU cytotoxic effects by downregulating the expression of P-gp and ABCG2 that resulted in an increase in drug accumulation within treated cells [26]. Induction of sensitivity may also likely be attributed to the inhibition of EMT and NF-κB pathways mediated by oxymatrine as one of the many bioactive components contained in Sophora injection [92]. Similar resistance-reversing effects were observed in oxaliplatin-treated cells, suggesting the potential of Sophora in reversing MDR. Correspondingly, phytosterol β-sitosterol, although it has only been shown to reverse oxaliplatin resistance in oxaliplatin-resistant cells, it may also work to reverse 5-FU resistance due to its potential in MDR modulation through the downregulation of BCRP expression [299]. Natural phenol curcumin is the bioactive compound responsible for the bright yellow pigment of turmeric. It was demonstrated that 5-FU therapy, when combined with curcumin, exhibited increased activation of 5-FU-triggered apoptosis in 5-FU-resistant cells through the effective inhibition of the Wnt signaling pathway and EMT activity mediated by curcumin treatment [91]. Downstream effects by these plant-derived small molecules may also be achieved through the regulations of non-coding RNAs. Ethanolic extract of fruit spike Spica Prunellae has shown to enhance 5-FU sensitivity in colon cancer cells by upregulating the expression of miR-494, which in turn, downregulates TOP2α expression [195]. Meanwhile, fruit-originated lignan schizandrin A may enhance 5-FU chemosensitivity by upregulating miR-195 that inhibits the PI3K/AKT and NF-κB signaling pathways [300]. Additionally, phenolic compound Kaempferol may work to impede the production of ROS and modulate the expression of JAK/STAT3/MAPK/PI3K/AKT, and NF-κB to reverse 5-FU resistance [28]. Recent studies on other plant-derived small molecules include the traditional Chinese medicine herbal formula Huang Qin Ge Gen Tang that modulates the E2F1/TS pathway, green tea extracted polyphenol epigallocatechin gallate that upregulates miR-155 to suppress MDR1 expression, β-elemene that can induce pro-death autophagy, and xanthonoid α-mangostin that have been successful in enhancing 5-FU cytotoxicity in colon cancer cells [59,81,301,302].

### 5.3. Non-Coding RNAs Regulators

In ovarian cancer cells, lncRNA taurine upregulated 1 (TUG1) overexpression has been implicated in the promotion of cellular proliferation and inhibition of apoptosis, suggesting its plausible involvement to also mediate tumorigenesis and chemoresistance in colon cancer cells [303]. Indeed, the knockdown of TUG1 has demonstrated enhanced 5-FU chemosensitivity via the downregulation of TYMS expression in colon cancer cells [228]. Similarly, knockdowns of PVT1, colon cancer-associated transcript 1 (CCAT1), and X-inactive specific transcript (XIST) in 5-FU-resistant colon cancer cells have also resulted in the successful restoration of 5-FU sensitivity [304,305,306]. Growing evidence has come to suggest lncRNAs exerting their transcriptional effects through functioning as competing endogenous RNAs (ceRNAs) by competitively binding onto miRNA sequence sites [307]. In TUG1-mediated resistance, increased TYMS expression was achieved through the suppression of miR-197-3p via ceRNA sponging, while in CCAT1-mediated resistance, a significant decrease in miR-218, miR-143, and miR-153 expression have been observed. H19 imprinted maternally-expressed transcript (H19) is another lncRNA reported to induce 5-FU resistance. Instead of regulating TYMS, H19 may sponge miR-194-5p to trigger the autophagy process via the SIRT1 enzyme [308]. Effective inhibition of these lncRNAs can be achieved through small interfering RNAs (siRNAs), RNA destabilizing elements (RDEs), antisense oligonucleotides (ASOs), and ribozymes; all of which had been demonstrated to target lncRNAs with high specificity [309,310]. In fact, recently, patisiran represents the world’s first siRNA-based drug that is FDA-approved for the treatment of hereditary transthyretin-mediated amyloidosis via transthyretin (TTR) gene silencing [311]. Additionally, recent advancement in miRNA therapy that saw the successful completion of phase II clinical trial for miR-122 antagonist as an effective antiviral agent against hepatitis C virus (HCV) infection may also be exploited to achieve enhanced 5-FU chemosensitivity [312]. Perhaps, microRNAs previously discussed to be upregulated in 5-FU-resistant cells such as miR-27a, miR-587, miR-31, miR-543, and miR-106a can also be silenced via miRNA repressors for its therapeutic potentials. Meanwhile, microRNA mimics can be applied to overexpress downregulated miRNAs such as miR-361, miR-200c, miR-22, miR-224, and many others. MesomiR-1 and MRG-201 are examples of miRNA mimic currently in phase I of clinical trial for the treatment of malignant pleural mesothelioma and keloid scar tissue formation, respectively [313]. NcRNA delivery strategies in vivo for translational applications have also been looked at in recent years following promising effectiveness of ncRNAs in vitro studies. In their paper, Wang and colleagues (2019) have recognized the reported low bioavailability and transfection efficiency, as well as the occurrence of off-target effects associated with these nucleic acid drugs *in vivo*. They have underlined several approaches that may benefit to overcome these challenges [314]. They include the use of nanoparticle and oncolytic adenovirus delivery systems, as well as the structured modification to subjected ncRNAs. For instance, engineered-exosomes for targeted co-delivery of miR-21 inhibitor have demonstrated to be effective in reversing 5-FU resistance in vivo [315].

### 5.4. Targeted Immunotherapy

It has been established that TILs are involved in chemotherapy response as elevated levels of chemokine CXCL-13 is determined in sera of 5-FU-resistant patients [145]. Knockdown of CXCL-13 resulted in re-sensitization of tumor cells with a significant increase in the number of B-cell infiltrating into the tumor cells. Furthermore, these resistant cells also highly-express chemokine CCL20 that promotes the recruitment of regulatory T cells (Tregs) via FOXO1/CEBPB/NF-κB signaling, suggesting that CCL20 inhibition may promote enhanced 5-FU chemosensitivity [151]. Indeed, dendritic cell therapy may appear as a useful strategy in this scenario as it provokes anti-tumor response through the increased number of dendritic cells (DCs) that may present tumor antigens to these lymphocytes. In one study, the use of 5-FU plus oxaliplatin with CD1d-MC38/α-GC tumor vaccine that promotes DC maturation had synergistically delayed tumor growth rate and increased the survival time of tumor-bearing mice [316]. Tumor cells may also escape immune cells through the secretion of immunosuppressive factors, such as TGF-β and IL-6, as well as through the recruitment of Tregs [152,158]. Recent studies have also demonstrated the upregulation of PD-L1 as the ligand for immune checkpoint programmed cell death-1 (PD-1) in 5-FU-treated cells promoted the abrogation of T-cell proliferation via the inhibition of PI3K/AKT signaling pathway [317,318]. Surprisingly, the use of PD-1 inhibitor Nivolumab alongside 5-FU, has instead promoted increased tumor cells tolerance against 5-FU [319].

## 6. Conclusions

Resistance to 5-FU, either intrinsic or acquired, can be attributed to various underlying mechanisms that may influence its cellular bioavailability, metabolism, and antitumor effects within tumor cells. These mechanisms are mostly driven by various aberrantly-expressed genes and proteins as the pro-survival response of tumor cells to tolerate 5-FU-induced cytotoxicity. Since tumor responses to 5-FU treatment may vary between individuals due to genetic heterogeneity and clonal evolution of tumor microenvironment, the practice of personalized medicine is perhaps the best way forward in the effort to overcome 5-FU resistance and improve 5-FU treatment response in colon cancer patients. The dysregulated tumor response to 5-FU treatment can be profiled in each patient and can be independently restored to regular function to overcome 5-FU resistance.

## Figures and Tables

**Table 1:** MiRNAs that mediate 5-FU resistance in colon cancer cells through the modulation of various pathways.

miRNA	Resistance Expression	Mechanism	Ref
miR-361	Underexpressed	Upregulation of ABCC10/5 expression regulated by FOXM1	[197]
miR-27a	Overexpressed	Modulation of DPYD expression	[198]
miR-200c	Underexpressed	Enhanced Bcl-2 expression	[199]
miR-587	Overexpressed	Downregulation of PPP2R1B expression that increases AKT phosphorylation and XIAP expression	[200]
miR-22	Underexpressed	Promotes autophagy via BTG1 upregulation	[201]
miR-224	Underexpressed	Suppressed apoptosis via repressed E2F activity	[94]
miR-375	Underexpressed	Upregulate TYMS expression	[202]
miR-31	Overexpressed	Downregulation of KANK1 that abrogate CXXC5-mediated apoptosis	[203]
miR-204	Underexpressed	Directly targets HGMA2 that activates the PI3K/Akt signaling pathway	[204]
miR-214	Underexpressed	Downregulation of HSP27	[205]
miR-543	Overexpressed	Upregulation of PTEN that activates the PI3K/AKT signaling pathway	[206]
miR-15b	Underexpressed	Downregulations of NF-κB1 and IKK-α targets	[207]
miR-106a	Overexpressed	Downregulation of TGFβR2 that promotes EMT and inhibit apoptosis	[208]
miR-206	Underexpressed	Enhanced Bcl-2 expression	[209]
miR-532	Underexpressed	Promotes Wnt/β-catenin signaling activation	[194]
miR-494	Underexpressed	Upregulation of TOP2α that facilitates DNA-damage repair	[195]
miR-125b	Underexpressed	Induction of Sp1/CD248 expression	[210]
miR-377	Underexpressed	Induction of BRD4 expression	[211]
miR-139	Underexpressed	Modulation of NOTCH1, Bcl-2, and AMFR expression	[212]
miR-27b	Underexpressed	Upregulation of ATG10 that promotes autophagy	[213]

**Table 2:** Recent evidence on the correlation of thymidylate synthase (TYMS), thymidine phosphorylase (TYMP), dihydropyrimidine dehydrogenase (DPD), and methylenetetrahydrofolate reductase (MTHFR) expression with 5-FU chemosensitivity in colon cancer cell lines and patients.

Enzyme	Resistance Mechanism	Recent Evidence	Outcome
Thymidylate synthase	TYMS overexpression via gene amplification through CNV and tandem repeats	Resistance in patients with 5′-UTR 2R/3G, 3C/3G, and 3G/3G polymorphisms [226]. Increase disease relapse in patients with 3′UTR ins/LOH polymorphism [227]. TYMS copy number gains in patients treated with 5-FU [7]. Increased TYMS expression in 5-FU-resistant colon cancer cell line [228]. TYMS knockdown enhances 5-FU chemosensitivity in colon cancer cell line [202].	Restored level of dTMP
Thymidine phosphorylase	TYMP suppression via loss-of-function	Meta-analysis: poor patients’ prognosis when TYMP is suppressed [229]. Increase disease recurrence in patients with TYMP underexpression [230]. Low TYMP expression is associated with reduced patients’ relapse-free survival (RFS) [231]. Low TYMP is associated with poor patient outcome [232]. Low TYMP is associated with shorter patients RFS and increased risk of disease recurrence [233]. TYMP upregulation is associated with enhanced 5-FU accumulation and response in colon cancer cell line [234].	Reduced intra-tumoral concentration of active 5-FU metabolite
Dihydropyrimidine dehydrogenase	DPD upregulation via polymorphism	DPD is upregulated in 5-FU-resistant mice [235]. DPD downregulation demonstrated anti-5-FU resistance in colon cancer cell line [236]. DPDI-1, ethynyluracil DPDI-2, S-1 DPDI-3, and BOF-A2 DPDI-4 was demonstrated as effective DPD inhibitors that can potentiate 5-FU [237]. DPD polymorphism profile may serve as an independent risk factor of 5-FU chemosensitivity [226]. DPD upregulation is observed in 5-FU-resistant mice [238].	
Methylenetetrahydrofolate reductase	MTHFR upregulation via polymorphism	Patients with rs1801131 point mutation are associated with shorter OS and disease-free survival (DFS) [239]. MTHFR 1298 A/A and heterozygous MTHFR 677 C/T genotype is associated with 5-FU resistance in colon cancer cell lines [240].	

**Table 3:** Heterogeneity in DPD expression can be contributed by various single nucleotide polymorphisms (SNPs) that leads to differing 5-FU response outcome in colon cancer patients.

SNP	Gene	Genotype	Outcome	Remark	Ref
c.1905 + 1	DYPD*2A	G > A	50% DPD reduction in heterozygous carrier <25% DPD reduction in homozygous carrier 40–80% 5-FU clearance in heterozygous carrier	0.1% in African American population 1% in Caucasian population 1.6% in European population	[15,264]
c.2846	D949V	A > T	30% DPD reduction in heterozygous carrier 39–59% DPD reduction in homozygous carrier 40–80% 5-FU clearance in heterozygous carrier	0.1% in African American population 1.1% in Caucasians 0.7% in European population	[265,266]
c.1679	DYPD*13	T > G	68% DPD reduction in heterozygous carrier <25% DPD reduction in homozygous carrier	0.07–0.1% in Caucasian population	[267]
c.1129–5923	HapB3	C > G	35% DPD reduction in heterozygous carrier 41–65% DPD reduction in homozygous carrier	4.7% in European population	[267,268]
c.1236		G > A			
DPYD*5	T85C	T > C	39% reduction in 5-FU efficiency	16% in late-stage patients	[226]
DPYD*9A	A1627G	AG + -GG	100% reduction in 5-FU efficiency	9% of late-stage patients	[226]

**Table 4:** Small molecule inhibitors that have been recently demonstrated to be capable in potentiating 5-FU chemosensitivity in colon cancer cells.

Small Molecule Inhibitor	Target	Mechanism	Ref
Depsipeptide	HDAC	Elevation of MHC class II expression and cellular apoptosis	[275]
Valproic acid (VPA)		Modulation of TYMS and TYMP expression	[276]
Suberanilohydroxamic acid (SAHA)		Decreased TYMS mRNA and protein expression	[288]
Cobimetinib	MEK	Decreased TYMS expression	[280]
Selumetinib		Abrogation of TK1 expression	[289]
U0126		Generation of more γH2AX foci, diminishing ERCC1 and TYMS expression	[282]
Thiostrepton	FOXM1	Suppression of TYMS expression and the regulation of TK-1 and TYMPS expression	[281]
Luminespib	HSP90	Downregulation of TYMS	[277,278]
Ganetespib		Not stated	[279]
Apatorsen	HSP27	Accelerated apoptosis	[290]
Ibrutinib	BTK	Inhibition of TGFB1 protective response and induction of pro-apoptotic E2F expression	[291]
AVL-292			
Trimethylglycine	STAT6	Increased E-cadherin marker and decreased ERCC1 expression	[292]
JTE-013	S1PR2	Downregulation of DPD expression	[236]
Gimeracil	DPD	Downregulation of DPD expression	[293]
Quinacrine	Nrf2	Increases the susceptibility of tumor cells to 5-FU under hypoxic conditions	[285]
Tipiracil	TYMP	Downregulations of TYMP	[118]
F5446	SUV39H1	Increased Fas expression and FasL-induced apoptosis	[293]
Aminooxyacetic acid (AOAA)	Hydrogen sulfide	Downregulations of TYMS and EREG expression	[294]
SLC-0111	Carbonic anhydrase IX	Not stated	[295]
AT7519	CDK	Not stated	[296]
Ribavirin	Eif4E	Increased cell cycle arrest at G_2_/M phase via increased cyclin B1, p-histone (Ser10), and Mad2 expression	[297]
Diethylaminobenzaldehyde (DEAB)	ALDH1	Not stated	[126]
